# Supplementary material for: Backward induction-based deep image search
Source: PLoS One. 2024 Sep 9;19(9):e0310098. doi: 10.1371/journal.pone.0310098 (PMC11383237; doi:10.1371/journal.pone.0310098)
Supplement: Attachment — Submitted filename: Response to Reviewer.docx [file pone.0310098.s002.docx]

**Journal: PLOS ONE
Paper Title: Backward Inductive Deep Neural Image Search
Manuscript ID: PONE-D-24-16658R1**

**EMID: f8c28b9d1eacdc24
Date: Jul 15, 2024**

**Response to Reviewer’s Comments**

The authors express their appreciation to the editor, academic editor, and reviewers for their insightful comments and recommendations, which have enhanced the quality of our manuscript. The manuscript has been updated based on these comments. Below, we list the detailed changes made to the manuscript and our responses to the questions, using "C" to denote Comment and “R” for Response.

**To Academic Editor**

C1: Please review your reference list to ensure that it is complete and correct. If you have cited papers that have been retracted, please include the rationale for doing so in the manuscript text, or remove these references and replace them with relevant current references. Any changes to the reference list should be mentioned in the rebuttal letter that accompanies your revised manuscript. If you need to cite a retracted article, indicate the article’s retracted status in the References list and also include a citation and full reference for the retraction notice.

R1:

Thank you for your advice on the completeness of the reference list. We reviewed and revised the reference information. The main changes we made are as follows: (We apologize for the untracked changes in the reference section, as we used Endnote for reference management.)

- Revised the journal/conference names to their official titles. (e.g. Association for the Advancement of Artificial Intelligence revised to AAAI Conference on Artificial Intelligence)
- Some papers have been updated on arXiv, resulting in multiple citation versions exists. We updated the citations to reflect the final versions of the papers.
- We searched and added the page numbers where the respective references are in the journal or proceedings.

We also carefully searched whether any of the references have been retracted. To verify the retraction status, we checked the respective journal websites and cross-checked with http://retractiondatabase.org/, www.webofscience.com, and [www.semanticscholar.org](http://www.semanticscholar.org). As a result, we confirmed that there are no retracted papers in the reference list. For papers published on arXiv, we confirmed that they are still publicly available. Please let us know if there are any issues with the references that we have not noticed, and we will make the necessary corrections.

**To Reviewer 1**

C1: I would suggest the title “Backward Induction-based Deep Image Search”. It is not mandatory to follow this suggestion. I leave it to the worthy Editor to check and accept the article.

R1: Thank you for your advice on revising the title of our paper. As you suggested, we have changed it to "Backward Induction-based Deep Image Search" to better emphasize our proposed method.
